# Supplementary figures and images for: Cloning, Heterologous Expression, and Antifungal Activity Evaluation of a Novel Truncated TasA Protein from Bacillus amyloliquefaciens BS-3
Source: Int J Mol Sci. 2025 Aug 4;26(15):7529. doi: 10.3390/ijms26157529 (PMC12347349; doi:10.3390/ijms26157529)

EQSANVNLNLKPGDK

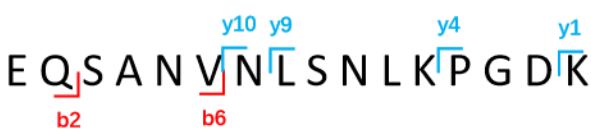

HFX1\_LFQ\_X0042250405\_GM001541.raw #10872 RT: 13.1202 min  
FTMS, 857.9437@hcd27.00, z=+2, Mono m/z=857.44312 Da, MH+=1713.87895 Da, Match Tol.=0.02 Da

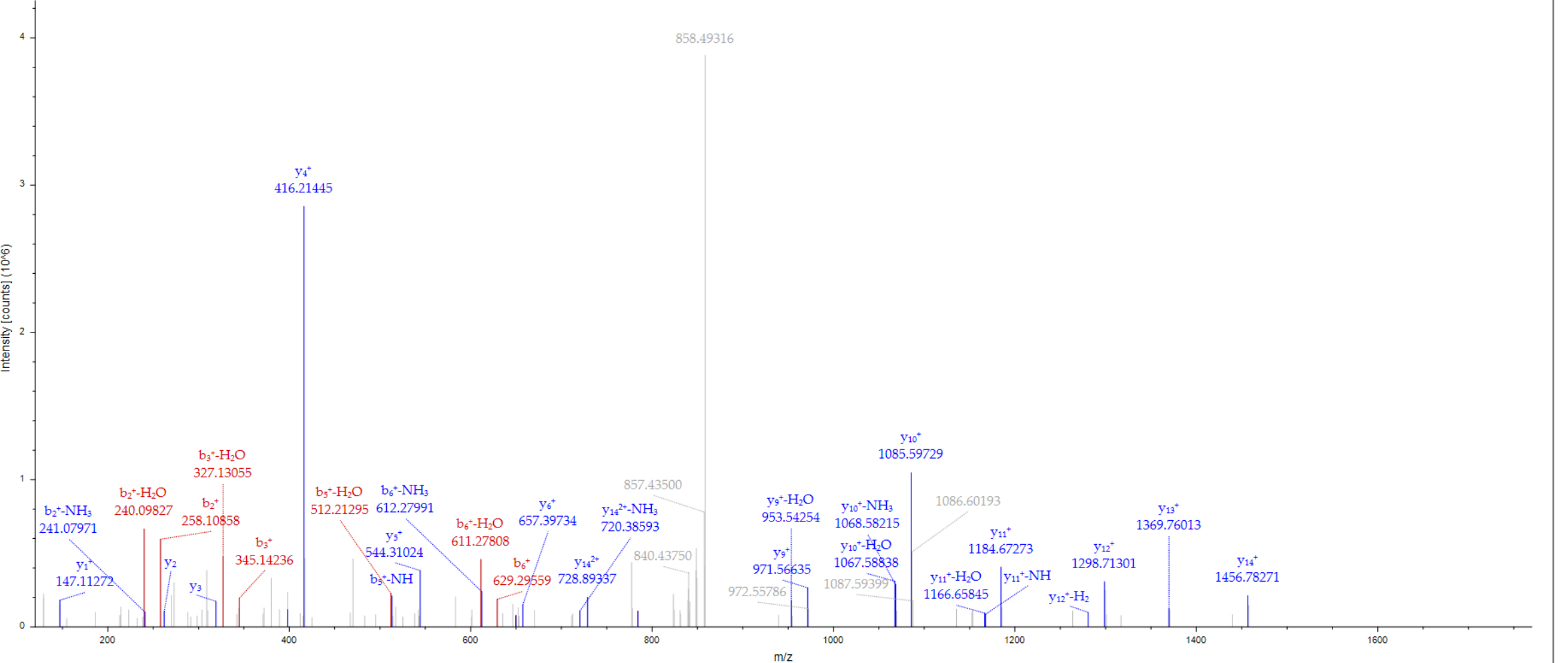

Supplement: Supplementary file 1 [file ijms-26-07529-s001.zip › Fig.S1.pdf]

HFX1\_LFQ\_X0042250405\_GM001541.raw #15092 RT: 16.3187 min  
FTMS, 470.7441@hcd27.00, z=+2, Mono m/z=470.74408 Da, MH+=940.48088 Da, Match Tol.=0.02 Da

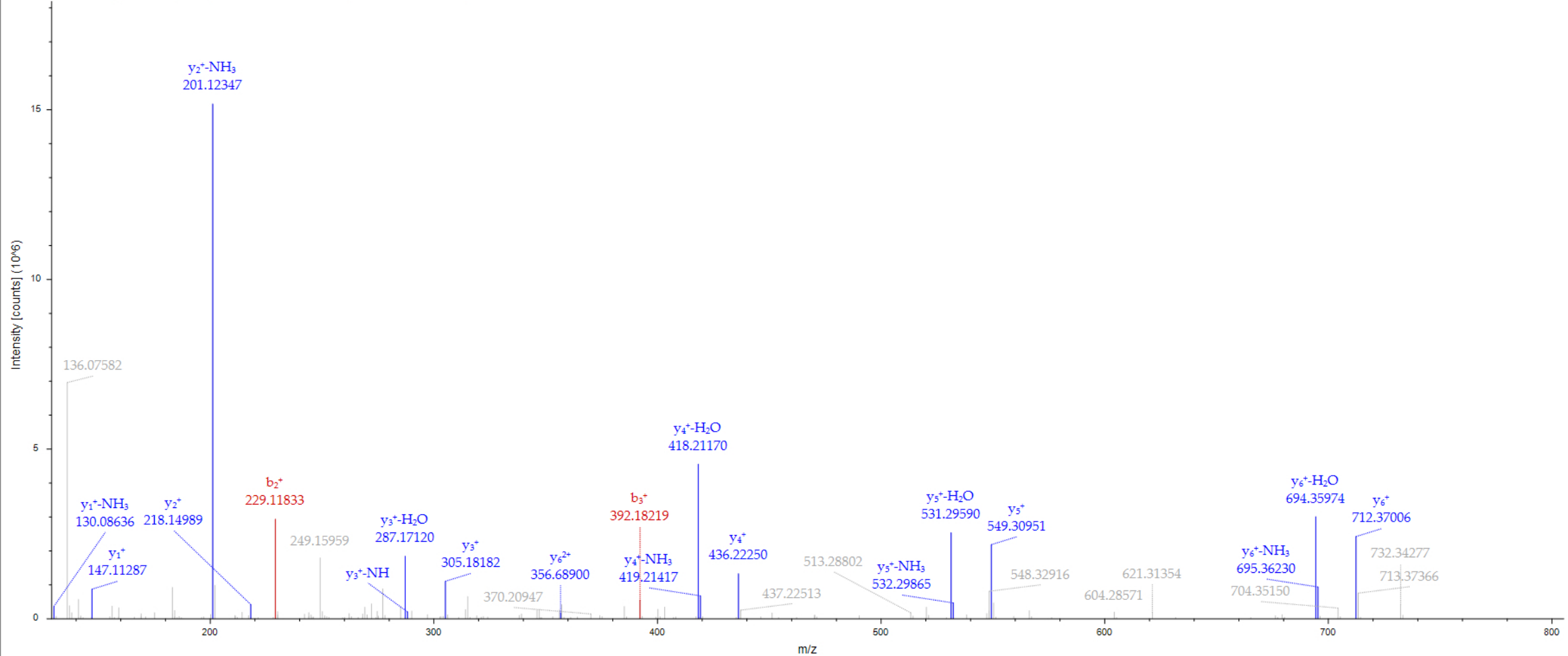

Supplement: Supplementary file 1 [file ijms-26-07529-s001.zip › Fig.S2.pdf]

AAAEAISILIR  
b2 b3 b4 b5

HFX1\_LFQ\_X0042250405\_GM001541.raw #17407 RT: 18.1524 min  
FTMS, 564.3431@hcd27.00, z=+2, Mono m/z=564.34308 Da, MH+=1127.67888 Da, Match Tol.=0.02 Da

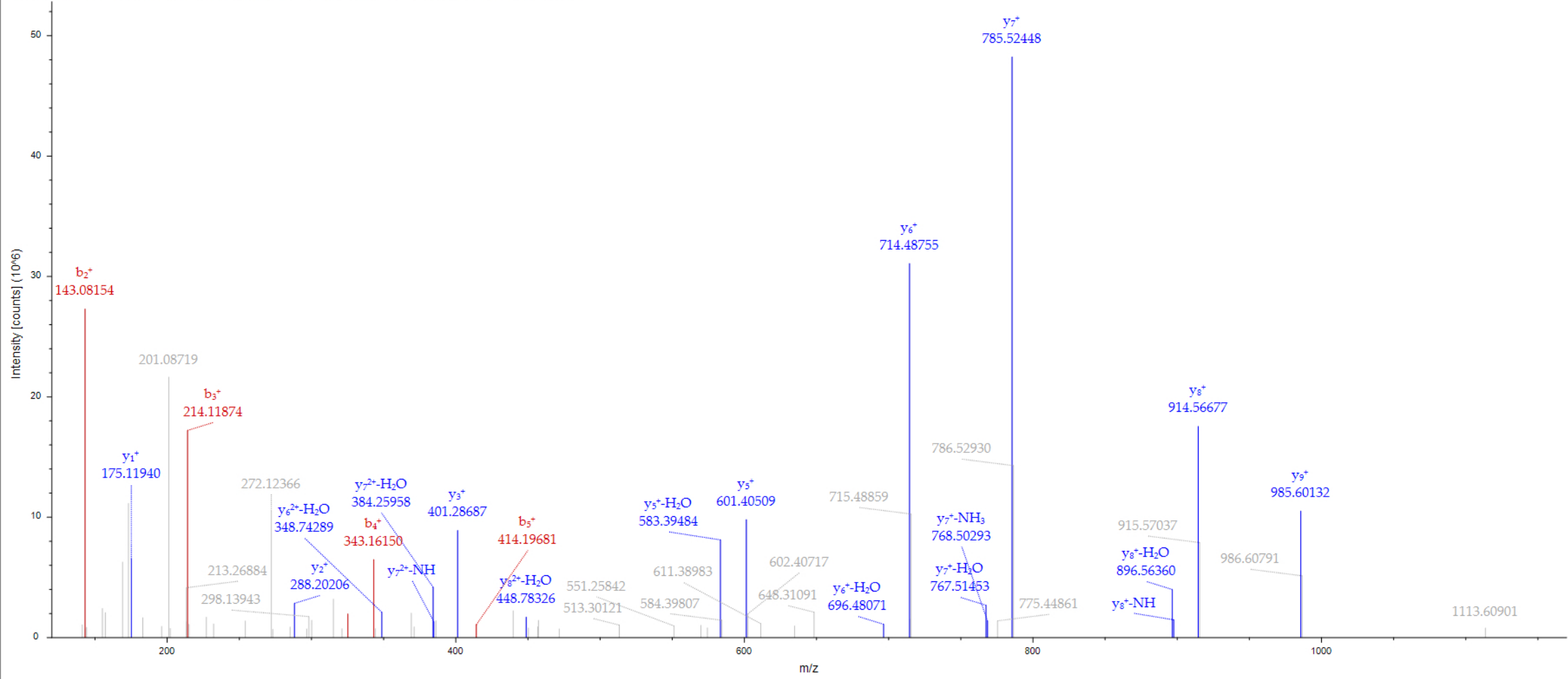

Supplement: Supplementary file 1 [file ijms-26-07529-s001.zip › Fig.S3.pdf]
